# Supplementary material for: Targeting FTO Suppresses Pancreatic Carcinogenesis via Regulating Stem Cell Maintenance and EMT Pathway
Source: Cancers (Basel). 2022 Nov 30;14(23):5919. doi: 10.3390/cancers14235919 (PMC9737034; doi:10.3390/cancers14235919)
Supplement: Supplementary file 1 [file cancers-14-05919-s001.zip › cancers-1986659-supplementary.pdf]

cancers

MDPI

Supplementary Materials:

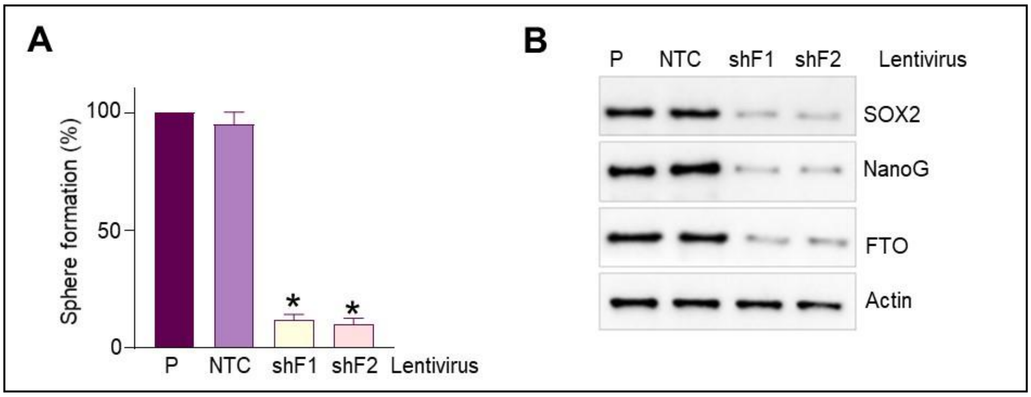

Figure S1. *FTO is required to maintain the self-renewal capacity of pancreatic cancer CSCs. (A)* Quantification of secondary spheres formed in the self-renewal assay. (B) protein expression of the markers of stem cell characteristics as analyzed by western blot in the protein extracts prepared from the secondary spheres generated from the parental, NTC, or shF1 and shF2 cells. Representative blots are shown.

Table S1. List of primers used in the study.

Genes	Forward primer	Reverse primer
FTO	5'- GCGGTGGCAGTGTACAGTTA -3'	5'-CCTGCCTTCGAGATGAGAGT-3'
E-cadherin	5′-ATTCTGATTCTGCTGCTCTTG-3′	5′-AGTAGTCATAGTCCTGGTCTT-3′
N-cadherin	5′- CTCCTATGAGTGGGAACAGGAACG-3′	5′-TTGGATCAATGTCATAATCAAGTGCTGTA-3′
Vimentin	5′-CCGGTGCAATCGTGATCTCTGGG-3′	5′-ATTCAAGTCTCAGCGGGCTC-3′
Fibronectin	5′-CTCCAAGTACCCCCTGAGGAA-3′	5′-CCAGGAGACTGTGAGCACTCC-3′
CD44	5′-CGGACACCATGGACAAGTTT-3′	5′-CCGTCCGAGAGATGCTGTAG-3′
NanoG	5'-CCGAAGAATAGCAATGGTGTGACG-3'	5'-AGGAGAATTTGGATGGAACTG C-3'
Sox2	5′-TCCCATCACCCACAGCAAATGA-3′	5′-TTTCTTGTCGGCATCGCGGTTT-3′
ALDH1	5′-AGCAGGAGTGTTTACCAAAGA-3′	5′-CCCAGTTCTCTTCCATTTCCAG-3′
CD133	5′-GCTCAGACTGGTAAATCCCC-3′	5′-GACTCGTTGCTGGTGAATTG-3′
β-actin	5′TCACCCACACTGTGCCCATCTACGA-3′	5′-CAGCGGAACCGCTCATTGCCAATGG-3′
